# Anti-Amyloidogenic Effects of *Metasequoia glyptostroboides* Fruits and Its Active Constituents

**DOI:** 10.3390/molecules28031017

**Published:** 2023-01-19

**Authors:** Ji-Yun Yeo, Seul Lee, Min Sung Ko, Chung Hyun Lee, Jee Yeon Choi, Kwang Woo Hwang, So-Young Park

**Affiliations:** 1 Laboratory of Pharmacognosy, College of Pharmacy, Dankook University, 119, Dandae-ro, Dongnam-gu, Cheonan-si 31116, Republic of Korea; 2 Host Defense Modulation Laboratory, College of Pharmacy, Chung-Ang University, Seoul 06974, Republic of Korea

**Keywords:** *Metasequoia glyptostroboides* fruits, beta-amyloid, Thioflavin T assay, sandaracopimarinol, sandaracopimaradien-19-ol, PC12 cells

## Abstract

Alzheimer’s disease (AD) is a serious neurodegenerative brain disease that interferes with daily life. The accumulation of beta-amyloid (Aβ), along with oxidative stress-inducing neurocellular apoptosis, has been considered one of the causes of AD. Thus, the purpose of this study is to find natural products that can reduce Aβ accumulation. The ethanol extract of *Metasequoia glyptostroboides* Hu & Cheng fruits (Cupressaceae) significantly reduced the aggregation of Aβ into oligomers and fibrils determined by Thioflavin T (ThT) assay. The solvent-partitioned ethyl acetate layer was further separated based on the bioassay-guided isolation method combined with the ThT assay. As a result, five compounds were isolated and elucidated as taxoquinone (**1**), sugiol (**2**), suginal (**3**), sandaracopimarinol (**4**), and sandaracopimaradien-19-ol (**5**) by comparing NMR data with references. All the compounds significantly reduced the aggregation of Aβ and enhanced the disaggregation of pre-formed Aβ aggregates in a dose-dependent manner. Furthermore, the inhibition of Aβ aggregation by the compounds protected PC12 cells from Aβ aggregate-induced toxicity. Among the five compounds, sandaracopimarinol (**4**) and sandaracopimaradien-19-ol (**5**) were the most effective. These results suggest that *M. glyptostroboides* and isolated five compounds have a potential for further study to be developed as anti-AD agents.

## 1. Introduction

As the proportion of the elderly population increases, dementia, which exhibits various cognitive impairments, becomes an important social and economic problem. It is predicted that 1 out of 85 people will develop dementia by the year 2050 [1]. Alzheimer’s disease (AD), which accounts for 60–70% of dementia in the elderly, is one type of neurodegenerative disease, and the representative symptom of AD is cognitive decline. Although the pathologic mechanisms of AD are not clearly elucidated, the pathologic hallmarks of AD are considered as the accumulation of beta-amyloid (Aβ) proteins in the form of senile plaques, the entanglement of hyperphosphorylated tau proteins in the form of neurofibrillary tangles, and loss of specific neurons [2,3,4]. Among them, the accumulation of Aβ protein into senile plaques is reported to impair cognitive function by disrupting the endoplasmic reticulum, insulin signaling, and mitochondrial functions, which are key signaling systems in cells [5,6,7].

Aβ is a protein produced by the proteolytic cleavage of amyloid precursor protein (APP) by the proteases called β- and γ-secretases. The cleavage of APP by β-secretase produces the large N-terminal ectodomain of APP (sAPPβ) and the membrane-bound C-terminal fragment, C99. The subsequent cleavage of C99 by γ-secretase eventually produces Aβ, which consists of 36–43 amino acid residues [5,8]. Aβ_42_, which consists of 42 amino acids, is known to be the most toxic. Aβ produced from APP accumulates extracellular compartments to form Aβ oligomers and fibrils, which cause the neurodegeneration, leading to the onset of AD symptoms [9,10].

Despite the efforts to discover the mechanisms of AD pathology, the exact causes of AD are not elucidated, nor are there suitable treatments available. Therefore, the experts mentioned that the prevention of AD could be the best treatment [11,12]. Previously, our lab has searched for various natural products, which can inhibit the production of Aβ and the subsequent aggregation to oligomers and fibrils. Among the many natural products tested, the extract of *Metasequoia glyptostroboides* Hu & Cheng fruits showed good inhibitory activity against Aβ aggregation. *M. glyptostroboides* is a deciduous coniferous tree belonging to the Cupressaceae family, distributed in many regions such as Europe, East Asia, and North America. It has feathery leaves and conical reddish-brown fruits [13]. Until now, the biological activities of *M. glyptostroboides* such as anti-bacterial, antioxidant, and anti-fungal effects have been reported [14,15]. It has also been used as a natural ingredient to maintain the quality of food as well as cooked food. However, *M. glyptostroboides* have not been studied for their effects on AD. Therefore, in this study, the ethanol extract of *M. glyptostroboides* fruits was tested for the potential inhibitory effects on Aβ aggregation with Thioflavin T (ThT) assay. In order to isolate active constituents that inhibit Aβ aggregation, a bioassay-guided isolation method using diverse column chromatographies (CCs) was employed. The structures of isolated compounds were elucidated by the comparison of NMR data with the references. The inhibitory effect of isolated compounds against Aβ aggregation and the protection of PC12 cells from Aβ aggregate-induced toxicity were determined by ThT and MTT assays, respectively.

## 2. Results

### 2.1. Inhibitory Effect of M. glyptostroboides Ethanol Extract and Solvent-Partitioned Fractions on Aβ Aggregation

**Figure 1:**
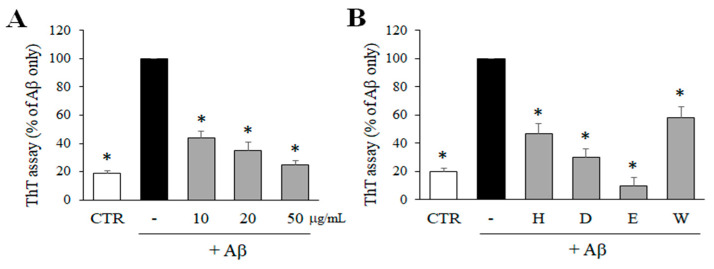
Effects of the ethanol extract and solvent-partitioned fractions on Aβ aggregation. (**A**) Aβ was incubated with 10, 20, and 50 µg/mL of *M. glyptostroboides* ethanol extract for 24 h, and ThT assay was performed to determine Aβ aggregation. (**B**) The 4 solvent-partitioned fractions at 50 µg/mL were tested for the effects on the aggregation of Aβ (H: *n*-hexane, D: dichloromethane, E: rthyl acetate, W: water). In case of control group, dimethyl sulfoxide (DMSO) was added instead of test samples. After 24 h incubation, the Aβ aggregation was determined by ThT assay. All data represent the mean ± SD of three different experiments. * *p* < 0.05, significantly different from Aβ only group.

In order to determine the inhibitory effects of *M. glyptostroboides* extract on Aβ aggregation, the level of Aβ aggregation was measured by ThT assay. As shown in Figure 1A, ethanol extract of *M. glyptostroboides* significantly reduced the aggregation of Aβ in a dose-dependent manner. Particularly, 50 μg/mL of the extract inhibited the aggregation of Aβ close to the control level. Then, the ethanol extract was partitioned based on solvent polarity, and four fractions including *n*-hexane, dichloromethane, ethyl acetate, and water fractions were obtained. These four fractions (50 μg/mL) were also tested to measure the inhibitory effect on the aggregation of Aβ. As a result, *n*-hexane, dichloromethane, ethyl acetate, and water fractions at 50 µg/mL significantly reduced the aggregation of Aβ down to 45.9, 28.4, 8.8, and 55.7%, respectively, compared to the Aβ only control group. Among the four fractions, the ethyl acetate fraction was the most active and was used for further experiments.

### 2.2. Isolation and Structure Elucidation of Compounds from M. glyptostroboides

**Figure 2:**
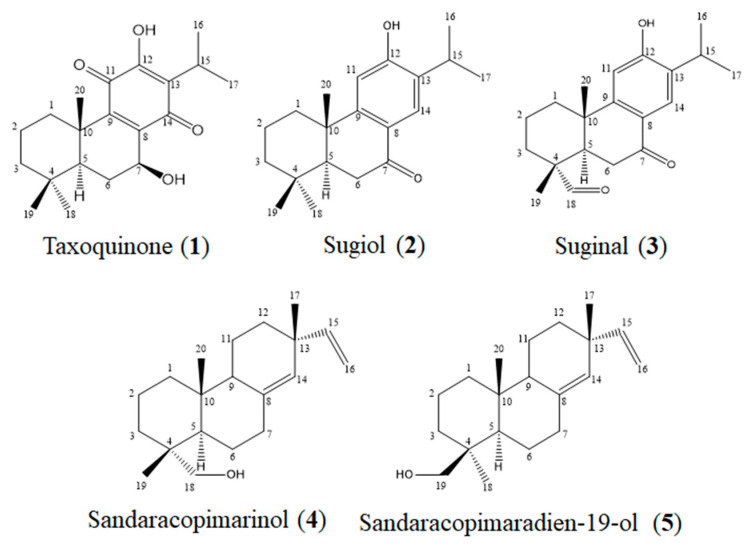
The chemical structures of the compounds isolated from *M. glyptostroboide*.

To isolate the active compounds that inhibit the aggregation of Aβ, the ethyl acetate fraction was fractionated into various open CCs and a Medium-Pressure Liquid Chromatography (MPLC) using silica gel as a stationary phase based on the bioassay-guided isolation method. As a result, five compounds were isolated from *M. glyptostroboides*. The structures of five compounds (compounds **1**–**5**) were elucidated based on the ^1^H and ^13^C-NMR data, and comparison with relevant references [16,17,18,19,20]. The isolated compounds were identified as taxoquinone (**1**), sugiol (**2**), suginal (**3**), sandaracopimarinol (**4**), and sandaracopimaradien-19-ol (**5**) (Figure 2).

### 2.3. Antioxidant Effect of Compounds Isolated from M. glyptostroboides

**Figure 3:**
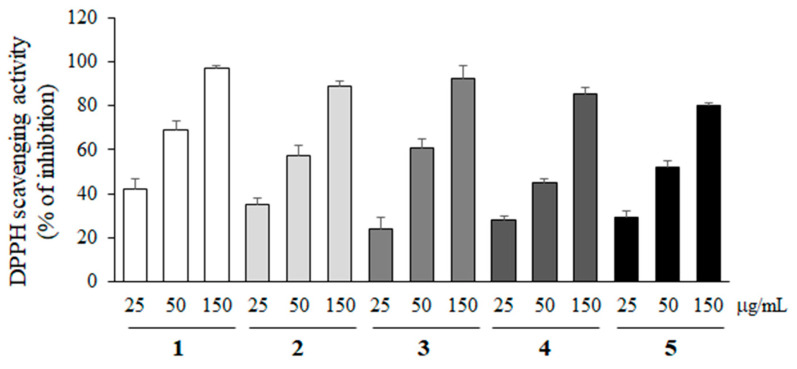
DPPH radical scavenging activity of compounds **1**–**5**. The antioxidant effects of compounds **1**–**5** were measured at 25, 50, and 150 µg/mL with DPPH free radicals. All data represent the mean ± SD of three different experiments.

2,2-Diphenyl-1-picryl-hydrazyl (DPPH) free radical scavenging activity of compounds **1**–**5** was measured at various concentrations (25, 50 and 150 µg/mL) to evaluate the antioxidant activity. As shown in Figure 3, all compounds showed the antioxidant activity, but their activities were not statistically different.

### 2.4. Inhibitory Effect of Compounds **1**–**5** on Aβ Aggregation

**Figure 4:**
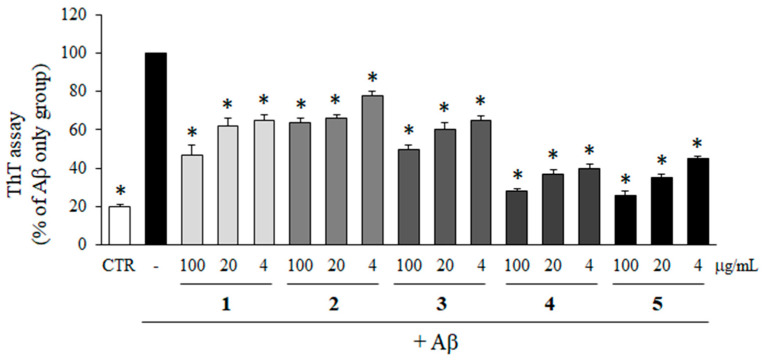
Effects of compounds isolated from *M. glyptostroboides* on Aβ aggregation. Aβ was incubated with 4, 20 and 100 µg/mL of compounds **1**–**5**. In case of control group, DMSO was added instead of test samples. After 24 h, the aggregation of Aβ was determined by ThT assay. All data represents the mean ± SD of three different experiments. * *p* < 0.05, significantly different from Aβ only group.

To determine the inhibitory effects of compounds **1**–**5** isolated from *M. glyptostroboides* on the aggregation of Aβ, ThT assay was performed. As shown in Figure 4, all of the compounds significantly inhibited the aggregation of Aβ. In particular, compounds **4** and **5** at 100 µg/mL reduced Aβ aggregation down to 26.9 and 25.4% compared to Aβ only group, which was close to the control group without Aβ. In addition, Aβ aggregations were reduced to 37.4 and 43.9% by the treatment with compounds **4** and **5** at 4 µg/mL. The inhibitory activities of compounds **4** and **5** at 4 µg/mL on Aβ aggregation were higher than compounds **1**, **2**, and **3** at 100 µg/mL which were 47.9, 66.8, and 54.8%, respectively.

### 2.5. Enhancement of Aβ Disaggregation by Compounds **1**–**5**

**Figure 5:**
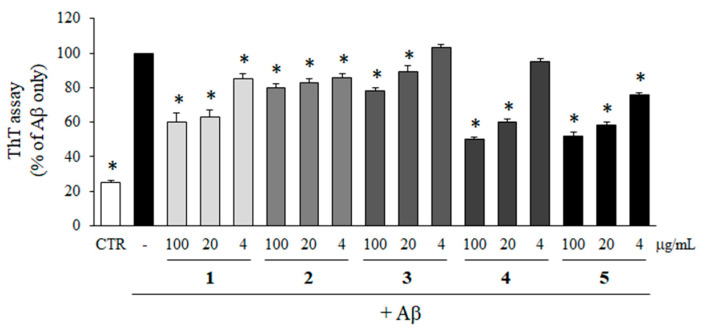
Activity of compounds **1**–**5** on the disaggregation of Aβ. Pre-aggregated Aβ in advance was incubated with 4, 20, and 100 µg/mL of compounds **1**–**5** for 24 h. In case of control group, DMSO was added instead of test samples. The Aβ disaggregation was determined by ThT assay. All data represent the mean ± SD of three different experiments. * *p* < 0.05, significantly different from Aβ only group.

To test the effects of the isolated compounds on the disaggregation of pre-aggregated Aβ, pre-aggregated Aβ in advance was incubated for 1 day with compounds **1**–**5**, and then the level of Aβ aggregation was detected by ThT assay. All of the compounds exhibited a positive effect on Aβ disaggregation in a dose-dependent manner. In particular, compounds **4** and **5** efficiently disaggregated the pre-aggregated Aβ and the level of aggregated Aβ was down to 50.0 and 53.2%, respectively, at 100 µg/mL compared to Aβ only group (Figure 5).

### 2.6. Inhibition of Aβ Aggregation by Compounds **1**–**5** Rescued the PC12 Cells from Aβ Aggregate-Induced Toxicity

**Figure 6:**
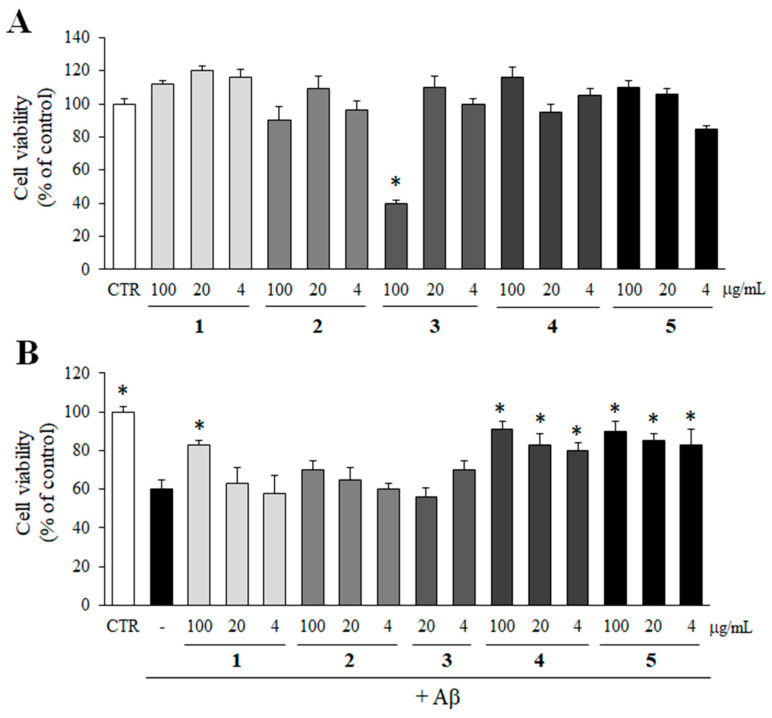
Inhibitory effect of compounds **1**–**5** on Aβ aggregate-induced toxicity. (**A**) The cytotoxicity of compounds **1**–**5** (100, 20, and 4 µg/mL) on PC12 cells was determined by MTT assay. (**B**) Pre-aggregated Aβ was incubated with compounds **1**–**5** for 24 h. In case of control group, DMSO was added instead of test samples. Then, PC12 cells were treated with the mixture of Aβ and compounds **1**–**5** for additional 24 h, and the viability of the cells was determined by MTT assay. All data represent the mean ± SD of three different experiments. * *p* < 0.05, significantly different from Aβ only group.

The possible cytotoxicity of compounds **1**–**5** on PC12 cells was determined by MTT assay. Compounds **1**–**5** were not cytotoxic to PC12 cells up to 100 µg/mL except compound **3** (Figure 6A). Compound **3** reduced the viability of PC12 cells only at 100 µg/mL. Thus, compound **3** at 100 µg/mL was excluded from the further study.

Then, an MTT assay was performed to observe whether the inhibition of Aβ aggregation by compounds **1**–**5** could rescue PC12 cells from Aβ aggregate-induced toxicity. As shown in Figure 6B, compounds **4** and **5**, which exhibited efficient inhibitory effects on Aβ aggregation in Figure 5, significantly increased the viability of cells from Aβ aggregate-induced toxicity at all concentrations. In addition, compound **1** at 100 µg/mL also protected the cells from Aβ aggregate-induced toxicity.

## 3. Discussion

Aβ, which is produced by proteolytic fragmentation of APP by β- and γ-secretases is considered one of the major causes of AD. In particular, over-produced Aβ aggregates into oligomers and fibrils, and Aβ aggregates are known to cause neurotoxicity and neuronal loss because of the increased mitochondrial dysfunction and ER stress [4,5,8]. In this study, the extract of *M. glyptostroboides* fruits significantly decreased the production of neurotoxic Aβ aggregates. In addition, five diterpenoids were isolated as active constituents. Among them, sandaracopimarinol (**4**) and andaracopimaradien-19-ol (**5**) efficiently decreased the aggregation of Aβ and enhanced the disaggregation of Aβ aggregates. Furthermore, sandaracopimarinol (**4**) and andaracopimaradien-19-ol (**5**) rescued the PC12 cells from Aβ aggregate-induced toxicity by inhibiting Aβ aggregation.

*M. glyptostroboides* is the only surviving large deciduous conifer known as a “living fossil” among the genus Metasequoia [16,17,18]. *M. glyptostroboides* has been successfully cultivated in nearly 50 countries, including Asia, Africa, Europe, and the United States [17,18,21]. *M. glyptostroboides* has been reported to have anti-bacterial, anti-fungal, antioxidant, and anti-inflammatory activities as well as neuroprotective effects [14,15]. It has been reported that *M. glyptostroboides* includes the largest group of terpenoids. The most frequently identified terpenoid is a monoterpenoid and the second is a sesquiterpenoid [22]. Dihydrohinokiflavone, a flavonoid family, was patented as an anticancer drug [13]. In addition, taxodone, metaseglyptorin A, metasequoia acid C, 12α-hydroxy-8,15-isopimaradien-18-oic acid, (-)-acora-2,4(14),8-trien-15-oic acid, and catechins were reported to be isolated [23].

The ethanol extract of *M. glyptostroboides* fruits was selected for further study because it efficiently inhibited Aβ aggregation determined by ThT assay. Then, the bioassay-guided isolation of ethanol extract of *M. glyptostroboides* fruits allowed to isolate five pure compounds. The structures of these compounds were elucidated based on ^1^H- and ^13^C-NMR data by comparison with references [16,17,18,19,20]. As a result, compounds **1**–**3** were identified as taxoquinone, sugiol, and suginal, respectively, which are abietane-type diterpenoids. The aromatic abietane structure is the most abundant naturally occurring abietane-type diterpenoid. Aromatic abietane-type diterpenoids, such as ferruginol, sclareol, isorosmanol, and carnorol have been reported to possess various biological properties, including anti-bacterial, anti-cancer, anti-fungal, anti-tumor, anti-viral, and anti-inflammatory activities [24,25,26,27]. However, the inhibitory effect of aromatic abietane-type diterpenoids on Aβ aggregation has not been reported, yet.

Taxoquinone (**1**) is known to be a natural anti-bacterial agent against a wide range of bacteria with little or no toxicity for centuries [28]. In addition, taxoquinone (**1**) has potent anti-diabetic and anti-melanin potential due to the inhibition of α-glucosidase and tyrosinase inhibition [16,28]. Sugiol (**2**) is also reported to efficiently inhibit α-glucosidase and tyrosinase and have potential anti-diabetic and anti-menalin effects [29]. Suginal (**3**) has been reported to have anti-bacterial activity [30]. It has a structure very similar to that of sugiol and has an additional carbonyl group.

In addition, compounds **4** and **5** are elucidated as sandaracopimarinol (**4**) and sandaracopimaradien-19-ol (**5**), pimarane-type diterpenoids. Pimarane-type diterpenoids are frequently produced from plants and fungi, but rarely produced from bacteria, insects, and marine organisms [31]. Pimarane-type diterpenoids are known to have important biological activities such as anti-bacterial, anti-fungal, anti-tumor, anti-viral, and cytotoxic activity [31,32,33]. Sandaracopimarinol (**4**) is known to have strong anti-bacterial and antioxidant activity. Sandaracopimaradien-19-ol (**5**) is reported to have anti-bacterial activity [34]. Even though diterpenoids with a pimarane structure such as pimaradienoic acid and continentalic acid have been reported to have anti-Alzheimer’s activities by inhibiting Aβ aggregation [33], the inhibitory effects of sandaracopimarinol (**4**) and sandaracopimaradien-19-ol (**5**) against Aβ aggregation were first reported in this study.

Taken together, these results suggest that the extract of *M. glyptostroboides* fruits and five diterpenoids isolated from *M. glyptostroboides* including taxoquinone (**1**), sugiol (**2**), suginal (**3**), sandaracopimarinol (**4**), and sandaracopimaradien-19-ol (**5**) significantly reduced the aggregation of Aβ and enhanced the disaggregation of Aβ aggregates from oligomers and fibrils to monomers. Furthermore, sandaracopimarinol (**4**), and sandaracopimaradien-19-ol (**5**) protected the neuronal cells from Aβ aggregate-induced toxicity by reducing Aβ aggregation. Therefore, the extract of *M. glyptostroboides* fruits and active constituents has the potential to prevent AD by inhibiting Aβ aggregation and protecting the neuronal cells.

## 4. Materials and Methods

### 4.1. Materials

RPMI 1640 was purchased from Welgene (Daegu, Republic of Korea). Fetal bovine serum (FBS) and horse serum were purchased from Gibco (Carlsbad, CA, USA). Aβ_1-42_ was purchased from GL Biochem (Shanghai, China). ThT and DPPH were purchased from Sigma (Sigma-Aldrich, St. Louis, MO, USA). DMSO and 3-(4,5-dimethylthiazol-2-yl)-2,5-diphenyl-tetrazolium bromide (MTT) were purchased from Biosesang (Seongnam, Republic of Korea). All the solvents for the extraction and CCs were purchased from Samchun Pure Chemical (Gyeonggi-Do, Republic of Korea).Thin Layer Chromatography (TLC) plates were used including TLC silica gel 60 F254 (Merck, Frankfurt, Germany). Stationary phases of open CC were Silica gel Si 60 (70-230 mesh, Watchers, Toyko, Japan). 

### 4.2. Plant Material and Extraction

The fruits of *M. glyptostroboides* were collected from Dankook University (Cheonan, Republic of Korea) in January 2020. A voucher specimen has been deposited in the Pharmacognosy Laboratory of College of Pharmacy, Dankook University, Republic of Korea. The dried and pulverized *M. glyptostroboids* fruits (4 kg) were extracted three times with ethanol (30 L) under reflux for 24 h at room temperature. The ethanol filtrate was evaporated under vacuum to yield the ethanol extract (310 g). The extract was suspended in distilled water and then partitioned sequentially into *n*-hexane, dichloromethane, ethyl acetate, and water. Four fractions including *n*-hexane (115.2 g), dichloromethane (46.0 g), ethyl acetate (30.9 g), and water (115.8 g) fractions were obtained.

### 4.3. Isolation of Active Compounds

The ethyl acetate fraction was fractionated by open CC using silica gel as stationary phase with solvent mixture of *n*-hexane and acetone (100:1), and 10 subfractions (MGE1~MGE10) were obtained. The subfraction 8 (MGE8) was further fractionated by open silica CC with solvent mixture of *n*-hexane and acetone (100:5), and compound **1** (89.5 mg) and compound **2** (222.5 mg) were obtained as pure compounds.

The subfraction 3 (MGE3) was further fractionated by open CC using silica gel with solvent mixtures of *n*-hexane and acetone (50:1). Among 9 subfractions, MGE3-9 was chromatographed by open silica gel CC with solvent mixture of *n*-hexane and acetone (10:1) and 6 (MGE3-9-1~MGE3-9-6) subfractions were obtained. The subfraction 3-9-5 (MGE3-9-5) was chromatographed by open silica gel CC with solvent mixture of chloroform and methanol (100:1), and compound **3** (76.0 mg) was obtained as a pure compound.

The subfractions 6 and 7 (MGE6,7) were combined and applied to open CC using silica gel with solvent mixture of dichloromethane and ethyl acetate (1:0~1:1), and 9 fractions (MGE6,7-1 ~ MGE6,7-9) were obtained. The subfraction 6,7-4 (MGE6,7-4) was further chromatographed and purified by silica gel CC with solvent mixture of chloroform and methanol (1:0 ~ 1:1) to give compound **4** (55.0 mg).

The subfraction 4 (MGE4) was chromatographed by MPLC (Isolera One, Biotage, Republic of Korea) using silica gel with solvent mixture of *n*-hexane and acetone (20:1), and 6 fractions (MGE4-1~MGE4-6) were obtained. Subfractions 4-2 (MGE4-2) were chromatographed and purified by silica gel CC with solvent mixture of *n*-hexane and acetone (2:1), and compound **5** was obtained as a pure compound (16.7 mg).

Compound **1** (Taxoquinone) ^1^H-NMR (CDCl_3_, 400 MHz): *δ* 4.77 (1 H, ddd, *J* = 2.2, 7.4, 9.8 Hz, H-7), 3.80 (1 H, d, *J* = 2.2 Hz, 7-OH), 3.13 (1 H, sept, *J* = 7.1 Hz, H-15), 2.65 (1 H, br. d, *J* = 13.2 Hz, H-1b), 2.18 (1 H, dd, *J* = 7.4, 12.5 Hz, H-6b), 1.34–1.80 (1 H, m, H-2/3/5), 1.32 (3 H, s, H-20), 1.20 (3 H, d, *J* = 7.2 Hz, H-17), 1.18 (3 H, d, *J* = 6.8 Hz, H-16), 0.91 (3 H, s, H-18), 0.90 (3 H, s, H-19). ^13^C-NMR (CDCl_3_, 100 MHz): *δ* 189.6 (C-14), 183.7 (C-11), 150.9 (C-12), 147.9 (C-9), 144.2 (C-8), 124.2 (C-13), 68.0 (C-7), 48.8 (C-5), 40.9 (C-3), 39.4 (C-10), 36.0 (C-1), 33.2 (C-18), 33.1 (C-4), 26.1 (C-6), 23.9 (C-15), 21.6 (C-19), 19.8 (C-16), 19.8 (C-17), 19.8 (C-20), 18.6 (C-2).

Compound **2** (Sugiol) ^1^H-NMR (DMSO, 400 MHz): *δ* 7.61 (1 H, s, H-14), 6.75 (1 H, s, H-11), 3.13 (1 H, sept, *J* = 6.9 Hz, H-15), 2.49 (1 H, dd, *J* = 17.6 Hz, H-6), 2.13 (1 H, br. d, *J* = 12.8 Hz, H-1), 1.75 (1 H, dd, *J* = 13.6 Hz), 1.34–1.80 (1 H, m, H-2/3), 1.13 (3 H, s, H-20), 1.12 (3 H, d, *J* = 6.8 Hz, H-17), 1.11 (3 H, d, *J* = 6.8 Hz, H-16). 0.90 (3 H, s, H-19), 0.85 (3 H, s, H-18). ^13^C-NMR (DMSO, 100 MHz): *δ* 197.0 (C-7), 160.6 (C-12), 156.3 (C-9), 133.0 (C-13), 125.5 (C-14), 123.1 (C-8), 109.8 (C-11), 49.6 (C-5), 41.4 (C-3), 38.0 (C-1), 38.0 (C-10), 36.1 (C-6), 33.4 (C-4), 32.8 (C-18), 26.6 (C-15), 23.6 (C-20), 22.9 (C-16), 22.7 (C-17), 21.7 (C-19), 19.0 (C-2).

Compound **3** (Suginal) ^1^H-NMR (CDCl_3_, 400 MHz): *δ* 9.88 (1 H, d, *J* = 3.0 Hz, CHO), 7.81 (1H, s, H-14), 6.92 (1 H, s, H-11), 3.16 (1 H, sept, *J* = 6.8 Hz, H-15), 1.80–2.08 (2 H, m, H-6), 1.50–1.91 (1 H, m, H-5), 1.24–1.71 (2 H, m, H-1/2), 1.45–1.60 (2 H, m, H-3), 1.20 (3 H, s, H-20), 1.01 (3 H, s, H-18), 1.26 (3 H, d, *J* = 6.8 Hz, H-17), 1.26 (3 H, d, *J* = 6.8 Hz, H-16). ^13^C-NMR (CDCl_3_, 100 MHz): *δ* 206.2 (C-18), 191.6 (C-7), 157.9 (C-9), 151.2 (C-12), 134.8 (C-13), 132.4 (C-14), 128.0 (C-8), 114.9 (C-11), 64.6 (C-5), 40.6 (C-4), 37.8 (C-1), 37.5 (C-10), 33.7 (C-6), 30.8 (C-3), 28.2 (C-15), 27.4 (C-19), 26.8 (C-20), 22.3 (C-16), 22.3 (C-17), 19.4 (C-2).

Compound **4** (Sandaracopimarinol) ^1^H-NMR (CDCl_3_, 400 MHz): *δ* 5.77 (1 H, dd, *J* = 17.3, 10.5 Hz, H-15), 5.21 (1 H, br. s, H-14), 4.90 (1 H, dd, *J* = 17.3, 1.5 Hz, H-16a), 4.87 (1 H, dd, *J* = 10.5, 1.5 Hz, H-16b), 3.40 (1 H, d, *J* = 10.9 Hz, H-18b), 3.12 (1 H, d, *J* = 10.9 Hz, H-18a), 2.23 (1 H, ddd, *J* = 11.8, 4.3, 1.8 Hz, H-7a), 2.06 (1 H, br. t, *J* = 11.8 Hz, H-7b), 1.75 (1 H, br. t, *J* = 7.5 Hz, H-9), 1.00–1.71 (2 H, m, H-1), 1.45–1.60 (2 H, m, H-2), 1.32–1.47 (2 H, m, H-3/12), 1.34 (1 H, m, H-5), 1.30–1.47 (2 H, m, H-6), 1.34–1.58 (2 H, m, H-11). ^13^C-NMR (CDCl_3_, 100 MHz): *δ* 149.2 (C-15), 137.1 (C-8), 128.8 (C-14), 110.1 (C-16), 72.3 (C-18), 50.6 (C-9), 48.0 (C-5), 39.0 (C-1), 38.2 (C-10), 37.9 (C-4), 37.5 (C-13), 35.8 (C-7), 35.6 (C-3), 34.7 (C-12), 26.1 (C-17), 22.5 (C-6), 18.9 (C-11), 18.4 (C-2), 18.1 (C-19), 15.7 (C-20).

Compound **5** (Sandaracopimaradien-19-ol) ^1^H-NMR (CDCl_3_, 400 MHz): *δ* 5.70 (1 H, dd, *J* = 17.1, 10.5 Hz, H-15), 4.90 (1 H, dd, *J* = 17.1, 2.1 Hz, H-16a), 4.95 (1 H, dd, *J* = 10.5, 2.1 Hz, H-16b), 5.12 (1 H, br. s, H-14), 3.82 (1 H, d, *J* = 10.0 Hz, H-19a), 3.42 (1 H, d, *J* = 10.0 Hz, H-19b), 2.31 (1 H, ddd, *J* = 13.8, 4.2, 2.4 Hz, H-7b), 2.02 (1 H, dt, *J* = 13.8, 4.2 Hz, H-7a), 1.83 (1 H, dd, *J* = 13.8, 5.1 Hz, H-3b), 1.75 (1 H, br. t, *J* = 7.5 Hz, H-9), 1.68 (1 H, m, H-6b), 1.66 (1 H, m, H-1b), 1.48 (1 H, m, H-11b), 1.44 (2 H, m, H-2), 1.33 (1 H, m, H-6a), 1.27 (1 H, m, H-11a), 0.97–1.27 (2 H, m, H-12), 1.03 (1 H, m, H-1a), 1.22 (2 H, m, H-12a), 1.03 (3 H, s, H-20), 0.98 (3 H, s, H-17), 0.98 (3 H, s, H-18), 0.97 (1 H, m, H-3a). ^13^C-NMR (CDCl_3_, 100 MHz): *δ* 149.1 (C-15), 136.8 (C-8), 128.9 (C-14), 110.1 (C-16), 73.2 (C-19), 50.7 (C-9), 48.8 (C-5), 38.9 (C-1), 38.2 (C-10), 37.5 (C-4), 36.7 (C-13), 36.1 (C-7), 35.8 (C-12), 34.7 (C-3), 26.0 (C-17), 22.6 (C-18), 21.1 (C-6), 18.9 (C-11), 18.3 (C-2), 15.7 (C-20).

### 4.4. ThT Assay

To quantify the aggregation of Aβ to oligomers and fibrils, ThT assay was performed. The Aβ_1-42_ was dissolved in DMSO at 1 mg/mL concentration and test samples were also dissolved in DMSO. To monitor the effects of test samples on the Aβ aggregation, 20 µM of Aβ_1-42_ was incubated with various concentrations of test samples at 37 °C for 24 h. For the control group, DMSO was used instead of test samples. Then, 3 µM of ThT was added, and fluorescence was measured after 30 min using an Emax precision microplate reader (Molecular Devices, CA, USA) with excitation at 442 nm and emission at 485 nm. The Aβ only was used as a control, and each assay was repeated 3 times.

To monitor the disaggregation effects of test samples on the pre-aggregated Aβ, the ThT assay was performed. Briefly, 20 µM of Aβ_1-42_ was pre-incubated at 37 °C for 24 h. After that, various concentrations of test samples or DMSO were added and incubated at 37 °C for additional 24 h. Then, 3 µM of ThT was added and fluorescence was measured after 30 min using an Emax precision microplate reader (Molecular Devices) with excitation at 442 nm and emission at 485 nm. The Aβ only was used as a control and each assay was performed in triplicate.

### 4.5. DPPH Assay

Antioxidant activity was measured using DPPH. The sample (10 µL) was mixed with 0.2 mM DPPH reagent (190 µL). The mixture was vortexed and kept in a dark place at 37 °C for 30 min. The change in absorbance was measured with an E-max precision microplate reader (Molecular Devices) at 540 nm. Ascorbic acid was used as a positive control. The inhibition rate was converted into a percentage of the difference between the absorbance values of a negative control treated with DMSO and the test samples. All experiments were repeated three times. Percent inhibition was calculated as (%) = (*A*_control_ − *A*_test_)/*A*_control_ * 100.

### 4.6. Cell Cultures

PC12 cells (rat pheochromocytoma cells) were obtained from Korea Cell Line Bank (Seoul, Republic of Korea) and grown in RPMI 1640 with 15% heat-inactivated horse serum and 5% heat-inactivated FBS. The cells were incubated in a humidified 5% CO_2_ at 37 °C.

### 4.7. MTT Assay

In order to determine the cytotoxicity of test samples, MTT assay was performed. PC12 cells were harvested from flasks and plated in 96-well plates with 6 × 10^4^ cells per well. Plates were incubated at 37 °C for 3 h to allow the cells to attach to the plates. Test samples were diluted with DMSO and added to individual wells. DMSO was added in case of control group. The plates were then incubated for an additional 24 h at 37 °C. The cell viability was determined using an MTT toxicity assay by adding 10 µL of 5 mg/mL MTT to each well. After 3 h incubation at 37 °C, 70 µL of the medium was gently removed, and then 70 µL of DMSO was added to each well. Plates were incubated at room temperature for 30 min to dissolve the MTT formazan crystals, and then the absorbance was measured at 540 nm using an E-max precision microplate reader (Molecular Devices). An average from 3 replicate wells was used for each sample, and each assay was performed in triplicate.

To investigate whether the inhibition of Aβ aggregation by test samples could rescue the cells from Aβ aggregate-induced toxicity, test samples (4, 20, and 100 µg/mL) and Aβ_1-42_ (10 µM) were mixed together in 96-well plates and incubated at 37 °C for 24 h to inhibit the formation of Aβ aggregates. For the control group, DMSO was added instead of test samples. The mixture was added to PC12 cells and incubated at 37 °C for an additional 24 h. The cell viability was determined using MTT assay. An average from 3 replicate wells was used for each sample, and each assay was performed in triplicate.

### 4.8. Statistical Analysis

All data in figures are expressed as mean ± SD of three different experiments. Data were analyzed using a two-tailed Student’s t-test in Microsoft Excel ver. 2016. Differences with a *p*-value smaller than 0.05 were considered statistically significant.

## Data Availability

Not applicable.

## References

[1] Hobert L.E., Weuve J., Scherr P.A., Evans D.A. (2013). Alzheimer disease in the United States (2010–2050) estimated using the 2010 census. Neurology.

[2] Lippens G., Sillen A., Landrieu I., Amniai L., Sibile N., Barbier P., Leroy A., Hanoulle X., Wieruszeski J.M. (2007). Tau aggregation in Alzheimer’s disease: What role for phosphorylation?. Prion.

[3] Volloch D., Olsen B., Rits S. (2020). Alzheimer’s Disease is Driven by Intraneuronally Retained Beta-Amyloid Produced in the AD-Specific, βAPP-Independent Pathway: Current Perspective and Experimental Models for Tomorrow. Ann. Integr. Mol. Med..

[4] Richard J.O., Wong P.C. (2011). Amyloid precursor protein processing and Alzheimer’s disease. Annu. Rev. Neurosci..

[5] Calabrò M., Rinaldi C., Santoro G., Crisafulli C. (2020). The biological pathways of Alzheimer disease: A review. AIMS Neurosci..

[6] Zetterberg H., Burnham S.C. (2019). Blood-based molecular biomarkers for Alzheimer’s disease. Mol. Brain.

[7] Mullins R.J., Diehl T.C., Chia C.W., Kapogiannis D. (2017). Insulin Resistance as a Link between Amyloid-Beta and Tau Pathologies in Alzheimer’s Disease. Front. Aging Neurosci..

[8] Botteri G., Salvadò L., Gumò A., Hamilton D.L., Meakin P.J., Montagut G., Ashford M.L.J., Victoria C.M., Sonia F.V., Vendrell J. (2018). The BACE1 product sAPPβ induces ER stress and inflammation and impairs insulin signaling. Metabolism.

[9] Scheltens P., Blennow K., Breteler M.M., de Strooper B., Frisoni G.B., Salloway S., Van der Flier W.M. (2021). Alzheimer’s disease. Lancet.

[10] Brookmeyer R., Abdalla N., Kawas C.H., Corrada M.M. (2018). Forecasting the prevalence of preclinical and clinical Alzheimer’s disease in the United States. Alzheimer’s Dement..

[11] Vingtdeux V., Hamdane M., Loyens A., Gele P., Drobeck H., Begard S., Marie C.G., Delacourte A., Jean C.B., Buee L. (2007). Alkalizing drugs induce accumulation of amyloid precursor protein by-products in luminal vesicles of multivesicular bodies. J. Biol. Chem..

[12] Butterfield D.A., Sultana R. (2011). Methionine-35 of aβ(1-42): Importance for oxidative stress in Alzheimer disease. J. Amino Acids.

[13] Juvik O.J., Nguyen X.H.T., Andersen H.L., Fossen T. (2016). Growing with dinosaurs: Natural products from the Cretaceous relict Metasequoia glyptostroboides Hu & Cheng-a molecular reservoir from the ancient world with potential in modern medicine. Phytochem. Rev..

[14] Wen C.T., Yan Y.Q., Lin F.D., Yang H., Jiang X.L., Li Y.P., Liu D.S., Gong X., Xing D.W., Qin S.Z. (2019). Diterpenoids and sesquiterpenoids from the stem bark of Metasequoia glyptostroboides. Phytochemistry.

[15] Yang C., Zhagn X., Wang T., Hu S., Zhou C., Zhang J., Wang Q. (2019). Phenotypic Plasticity in the Structure of Fine Adventitious *Metasequoia glyptostroboides* Roots Allows Adaption to Aquatic and Terrestrial Environments. Plants.

[16] Bajpai V.K., Park Y.H., Na M.K., Kang S.C. (2015). α-Glucosidase and tyrosinase inhibitory effects of an abietane type diterpenoid taxoquinone from *Metasequoia glyptostroboides*. BMC Complement. Altern. Med..

[17] Bajpai V.K., Sharma A., Kang S.C., Baek K.H. (2014). Antioxidant, lipid peroxidation inhibition and free radical scavenging efficacy of a diterpenoid compound sugiol isolated from *Metasequoia glyptostroboides*. Asian Pac. J. Trop. Med..

[18] Bajpai V.K., Kim N.H., Kim K.M., Kang S.C. (2016). Antiviral potential of a diterpenoid compound sugiol from *Metasequoia glyptostroboides*. Pak. J. Pharm. Sci..

[19] Morisawa J., Kim C.S., Kashiwagi T., Tebayashi S.I., Horiike M. (2002). Repellents in the Japanese cedar, *Cryptomeria japonica*, against the pill-bug, *Armadillidium vulgare*. Biosci. Biotechnol. Biochem..

[20] Wei W., Li Y., Li H., Wang L., Gao K. (2019). Phytotoxic Diterpenoids form Plants and Microorganisms. Chem. Biodivers..

[21] Chen F., Zhang L., Zong S., Xu S., Li X., Ye Y. (2014). Antioxidant Capacity and Proanthocyanidin Composition of the Bark of *Metasequoia glyptostroboides*. Evid. Based Complement. Alternat. Med..

[22] Dong L.B., He J., Wang Y.Y., Wu X.D., Deng X., Pan Z.H., Xu G., Peng L.Y., Zhao Y., Li Y. (2011). Terpenoids and norlignans from *Metasequoia glyptostroboides*. J. Nat. Prod..

[23] Lee. H., Oh C., Kim S., Dey D.K., Kim H.H., Bajpai V.K., Han Y.K., Huh Y.S. (2021). Metasequoia glyptostroboides potentiates anticancer effect against cervical cancer via intrinsic apoptosis pathway. Sci. Rep..

[24] Gonzalez M.A. (2015). Aromatic abietane diterpenoids: Their biological activity and synthesis. Nat. Prod. Rep..

[25] Habtemariam S. (2016). The Therapeutic Potential of Rosemary (*Rosmarinus officinalis*) Diterpenes for Alzheimer’s Disease. Evid. Based Complement. Alternat. Med..

[26] Gonzalez M.A. (2014). Synthetic derivatives of aromatic abietane diterpenoids and their biological activities. Eur. J. Med. Chem..

[27] Hjortness M.K., Riccardi L., Hongdusit A., Ruppe S., Zhao M., Kim E.Y., Zwart P.H., Sankaran B., Arthanari H., Sousa M.C. (2018). Abietane-Type Diterpenoids Inhibit Protein Tyrosine Phosphatases by Stabilizing an Inactive Enzyme Conformation. Biochemistry.

[28] Bajpai V.K., Na M., Kang S.C. (2010). The role of bioactive substances in controlling foodborne pathogens derived from *Metasequoia glyptostroboides* Miki ex Hu. Food Chem. Toxicol..

[29] Wang Y., Shi L.Y., Qi W.H., Yang J., Qi Y. (2017). Anticancer activity of sugiol against ovarian cancer cell line SKOV3 involves mitochondrial apoptosis, cell cycle arrest and blocking of the RAF/MEK/ERK signalling pathway. Arch. Med. Sci..

[30] Chao K.P., Hua K.F., Hsu H.Y., Su Y.C., Chang S.T. (2005). Anti-inflammatory activity of sugiol, a diterpene isolated from *Calocedrus formossana* bark. Planta Med..

[31] Porto T.S., Rangel R., Furtado N.A.J.C., de Carvalho T.C., Martins C.H.G., Veneziani R.C.S., Costa F.B.D., Vinholis A.H.C., Cunha W.R., Heleno V.C.G. (2009). Pimaranee-type diterpenes: Antimicrobial activity against oral pathogens. Molecules.

[32] Reveglia P., Cimmino A., Masi M., Nocera P., Berova N., Ellestad G., Evidente A. (2018). Pimarane diterpenes: Natural source, stereochemical configuration, and biological activity. Chirality.

[33] Jung H.A., Lee E.J., Kim J.S., Kang S.S., Lee J.H., Min B.S., Choi J.S. (2009). Cholinesterase and BACE1 inhibitory diterpenoids from Aralia cordata. Arch. Pharm. Res..

[34] Anaya A.L., Mata R., Sims J.J., Coloma A.G., Ortega R.C., Guadano A., Bautista B.E.H., Midland S.L., Rios R., Pompa A.G. (2003). Allelochemical potential of *Callicarpa acuminata*. J. Chem. Ecol..

